# Supplementary material for: Coming out in a harsh environment: a new genus and species for a land flatworm (Platyhelminthes: Tricladida) occurring in a ferruginous cave from the Brazilian savanna
Source: PeerJ. 2018 Dec 4;6:e6007. doi: 10.7717/peerj.6007 (PMC6284438; doi:10.7717/peerj.6007)
Supplement: Supplemental Information 1 — Supplementary Table 1. Measurements, in mm, of the holotype of Difroehlichia elenae sp. nov. Supplementary Table 2. Body height and cutaneous musculature in the median region of a transverse section of the pre-pharyngeal region, in micrometers, and ratio of the height of cutaneous musculature to the height of the body (mc:h index) of the holotype of Difroehlichia elenae sp. nov. [file peerj-06-6007-s001.docx]

**Supplementary Table 1.** Measurements, in mm, of the holotype of *Difroehlichia elenae* **sp. nov.** *: after fixation; ** length; DG: distance of gonopore from anterior end; DM: distance of mouth from anterior end; DMG: distance between mouth and gonopore; DPVP: distance between prostatic vesicle and pharyngeal pouch. The numbers given in parentheses represent the position relative to body length.

|  | Holotype |
| --- | --- |
| Length* | 13.5 |
| Width* | 1.5 |
| DM* | 8.5 (63) |
| DG* | 10.5 (78) |
| DMG* | 2 |
| DPVP* | 0.9 |
| Ovaries | 3 (22) |
| Anteriormost testes | 4 (30) |
| Posteriormost testes | 8.5 (63) |
| Prostatic vesicle** | 0.2 |
| Main male atrium** | 0.4 |
| Secundary male atrium** | 0.3 |
| Female atrium** | 0.3 |

**Supplementary Table 2.** Body height and cutaneous musculature in the median region of a transverse section of the pre-pharyngeal region, in micrometers, and ratio of the height of cutaneous musculature to the height of the body (mc:h index) of the holotype of *Difroehlichia elenae* **sp. nov.**

|  | Holotype |
| --- | --- |
| Dorsal cutaneous musculature | 11-12 |
| Ventral cutaneous musculature | 8-10 |
| Dorsal epidermis | 10 |
| Ventral epidermis | 6-15 |
| Body height | 610-770 |
| Mc:h (%) | 2.7-3.3 |
